# Real-world treatment of adult patients with Guillain-Barré syndrome over the last two decades

**DOI:** 10.1038/s41598-021-98501-y

**Published:** 2021-09-27

**Authors:** Jakob Rath, Gudrun Zulehner, Bernadette Schober, Anna Grisold, Martin Krenn, Hakan Cetin, Fritz Zimprich

**Affiliations:** Department of Neurology, Medical University of Vienna, Währinger Gürtel 18-20, 1090 Vienna, Austria

**Keywords:** Neurological disorders, Peripheral neuropathies

## Abstract

This study investigated treatment characteristics of Guillain-Barré syndrome (GBS) in a real-world setting between 2000 and 2019. We analyzed clinical improvement between nadir and last follow-up in patients with severe GBS (defined as having a GBS disability scale > 2 at nadir) and aimed to detect clinical factors associated with multiple treatments. We included 121 patients (74 male; median age 48 [IQR 35–60]) with sensorimotor (63%), pure motor (15%), pure sensory (10%) and localized GBS (6%) as well as Miller Fisher syndrome (6%). 44% of patients were severely affected. All but one patient received at least one immunomodulatory treatment with initially either intravenous immunoglobulins (88%), plasma exchange (10%) or corticosteroids (1%), and 25% of patients received more than one treatment. Severe GBS but not age, sex, GBS subtype or date of diagnosis was associated with higher odds to receive more than one treatment (OR 4.22; 95%CI 1.36–13.10; *p* = 0.01). Receiving multiple treatments had no adjusted effect (OR 1.30, 95%CI 0.31–5.40, *p* = 0.72) on clinical improvement between nadir and last follow-up in patients with severe GBS. This treatment practice did not change over the last 20 years.

## Introduction

The Guillain-Barré syndrome (GBS) is an acute immune-mediated neuropathy with a range of clinical subtypes^1,2^. The two pharmacological treatments with proven efficacy are intravenous immunoglobulins (IVIg)^3^ and plasma exchange (PE)^4^, which are both equally effective in shortening time to recovery and improving clinical outcome but not in reducing mortality^5,6^. A second IVIg course after PE or IVIg has not been associated with an additional benefit regarding outcome in non-randomized studies^6,7^. Recently, a placebo-controlled, randomized trial in patients with poor prognosis also failed to show a treatment effect of a second IVIg course and led to a higher number of adverse events^8^.

Nonetheless, a substantial proportion of patients receive a second treatment in clinical practice because of clinical deterioration, lack of response or treatment-related fluctuations (TRF; i.e., clinical worsening after initial stabilization or improvement)^9^. Moreover, distinguishing GBS with TRFs from acute onset chronic inflammatory demyelinating polyneuropathy (CIDP) is sometimes difficult^10^.

Most studies included only patients with moderate to severe classical GBS (i.e., unable to walk independently), and treatment benefits in patients with mild disease or Miller Fisher syndrome respectively other focal variants are poorly understood. However, real-word data on treatment strategies of GBS suggest that a substantial proportion of patients, including patients with mild disease or focal variants, received a sequential therapy with the treatment selection varying depending on geographic regions^9^.

In this study, we retrospectively analyzed whether treatment practice at a large tertiary care center in Austria changed over the course of the last two decades and investigated whether treatment with IVIg and PE was carried out according to current recommendations^1,11^. We specifically aimed to investigate the number and characteristics of GBS treatments and whether they changed over the course of the last two decades.

## Methods

### Patients

We retrospectively examined clinical data of patients diagnosed with an acute immune-mediated neuropathy at the Department of Neurology of the Medical University of Vienna between January 2000 and December 2019. The study was approved by the ethics committee of the Medical University of Vienna (Ec-Nr. 1927/2016 and Ec-Nr. 2251/2020). The requirement to obtain patient consent was waived for this retrospective study by the Ethics Committee of the Medical University of Vienna. The study was carried out in accordance with the World Medical Association Declaration of Helsinki and relevant local regulations.

### Patient data

We grouped patients clinically into sensorimotor, pure motor or pure sensory GBS as well as localized variants and Miller Fisher syndrome. Patients with classical GBS/MFS overlap were classified as GBS. We retrospectively calculated the Medical Research Council (MRC) sum score^12^ ranging from 0 (complete paralysis) to 60 (normal strength) at admission and the GBS disability scale^13,14^ ranging from 0 to 6 with higher scores indicating more severe disease at nadir and last follow-up (within 1 year after diagnosis) to evaluate clinical severity. Mild GBS was defined as a GBS disability scale of 0–2 and severe GBS as a GBS disability scale of 3–6. TRFs were defined as a clinical deterioration after initial stabilization or improvement^11^. Rajabally’s criteria were used to analyze nerve conduction studies^15^. Upper reference limits (URL) for CSF/serum albumin quotients (Qalb) were calculated according to Hegen et al.^16^ and for age-adjusted (by decade of age) total protein according to McCudden and colleagues^17^. Detection of ganglioside antibodies was carried out in sera of patients using Enzyme-linked immunosorbent assays (ELISAs). We analyzed patient charts with regard to the number and order of treatments and time to treatments from clinical onset. Additionally, we calculated the recommended ideal dose of IVIg (2 g/kg bodyweight) with self-reported body weight values at admission (available in 120/121 patients) and computed the difference between the ideal and the actually received dose (excluding patients whose IVIg treatment was stopped prematurely due to treatment switch to PE). For PE we extracted the number of plasma exchanges and the time span of treatment.

### Statistical analysis

For statistical analysis, SPSS 26 software package (IBM Corp. Released 2019. IBM SPSS Statistics for Macintosh, Version 26.0. Armonk, NY: IBM Corp) and R version 4.02 (R Core Team, 2020. R: A language and environment for statistical computing. R Foundation for Statistical Computing, Vienna, Austria) as well as R Studio version 1.3.959 (RStudio Team, 2020. RStudio: Integrated Development for R. RStudio, PBC, Boston, MA) were used.

Independent categorical variables were compared with the Chi-square test and continuous variables with the Mann–Whitney U Test. Multivariate logistic regression was used to evaluate dichotomized number of treatments of patients (i.e., no or one treatment vs. more than one treatment) with age as continuous covariate and sex, time of onset (grouped into four 5-year intervals between 2000 and 2019), clinical severity (mild vs. severe GBS) and GBS subgroups as categorical covariates including all 2-way interactions. Multivariate logistic regression was also used to investigate clinical improvement in patients with severe GBS at last follow-up compared to nadir (dichotomized as no improvement vs. improvement of at least one point on the ordinal GBS disability scale) with age and time to first treatment as continuous covariate and GBS subgroup, sex as well as number of treatments (dichotomized as zero or one vs. more than one) as categorical covariates including all 2-way interactions. Additionally, factors associated with poor outcome were analyzed with age as continuous and sex, GBS subtype and electrophysiological category according to Rajabally’s criteria^15^ as categorical covariates including all 2-way interactions. SPSS’s general linear model was used to test the effect of variables on the number of PE using the same covariates as in the multivariate logistic regression analysis and a full factorial model with Type III sum of squares. *P* ≤ 0.05 was considered statistically significant. Bonferroni correction was applied to adjust p-values for multiple comparisons.

### Ethics approval

This study was approved by the Ethics Committee of the Medical University of Vienna (Ec-Nr. 1927/2016 and Ec-Nr. 2251/2020). The requirement to obtain patient consent was waived for this retrospective study by the Ethics Committee of the Medical University of Vienna.

### Consent to participate

All authors read and approved the final manuscript.

## Results

We retrospectively investigated 129 patients, of whom eight patients initially treated at other hospitals were excluded because of missing data. The remaining 121 (74 male; median age 48, IQR 35–60) were analyzed. 63% had classic sensorimotor GBS, 15% pure motor GBS, 10% pure sensory GBS, 6% Miller Fisher syndrome and 6% a localized variant. 56% of the cohort had mild GBS whereas 44% were severely affected according to the GBS disability scale. Table 1 shows detailed baseline characteristics of all patients as well as for patients with no or one treatment vs. 2 or more treatments.

**Table 1:** Clinical characteristics.

	All patients (N = 121)	Patients with no or one treatment (N = 91)	Patients with multiple treatments (N = 30)	p-value
Sex	74 (61%) males; 47 (39%) females	53 (58%) males; 38 (42%) females	21 (70%) males; 9 (30%) females	0.25
Median age (IQR; range)	48 (35–60; 20–84)	47 (33–58; 20–84)	55.5 (39.5–66; 23–77)	0.04
**GBS subtype**				0.21
Classic sensorimotor	63% (76/121)	58% (53/91)	76% (23/30)	
Pure motor	15% (18/121)	16% (15/91)	10% (3/30)	
Pure sensory	10% (12/121)	13% (12/91)	0	
Miller Fisher syndrome	6% (7/121)	6% (5/91)	7% (2/30)	
Localized variant	6% (8/121)	7% (6/91)	7% (2/30)	
**Preceding infection**				0.36
Gastrointestinal	31% (37/121)	31% (28/91)	30% (9/30)	
Respiratory	15% (18/121)	12% (11/91)	23% (7/30)	
Other	13% (16/121)	15% (14/91)	7% (2/30)	
**NCS (Rajabally’s criteria)**				0.50
Demyelinating	28% (32/116)	28% (24/86)	27% (8/30)	
Axonal	23% (27/116)	21% (18/86)	30% (9/30)	
Equivocal	39% (45/116)	38% (33/86)	40% (12/30)	
Normal	10% (12/116)	13% (11/86)	3% (1/30)	
IgG GM 1 antibodies	13% (9/69)	18% (9/50)	0% (0/19)	0.05
IgG GQ1b antibodies	13% (8/60)	18% (7/40)	5% (1/20)	0.18
**CSF**				
Cell count/µL (IQR)	2 (1–4)	2 (1–4)	2 (2–4)	0.94
TP above age-adjusted URL	47% (57/116)	48% (41/86)	53% (16/30)	0.59
Qalb above age-adjust URL	41% (49/107)	46% (37/80)	44% (12/27)	0.87
Median MRC sum score at admission (IQR)	54 (45.5–59.5)	54 (48–60)	47 (38–56)	0.004*
**GBS disability scale at nadir**				< 0.001*
1	18% (21/121)	21% (19/91)	7% (2/30)	
2	39% (47/121)	44% (40/91)	23% (7/30)	
3	12% (15/121)	10% (9/91)	20% (6/30)	
4	22% (27/121)	22% (20/91)	23% (7/30)	
5	9% (11/121)	3% (3/91)	27% (8/30)	
6	0	0	0	

Clinical features of all patients as well as for patients with no or one treatment versus patients with multiple (2 or more) treatments as well as univariate comparison between the two groups are shown. MRC sum scores were calculated at admission and GBS disability scale at nadir. Upper reference limits for age-adjusted total protein were calculated according to McCudden et al.^17^ and for Qalb according to Hegen et al.^16^. CSF denotes cerebrospinal fluid, IQR interquartile range, MRC Medical Research Council, NCS nerve conduction study, Qalb CSF/serum albumin quotient and URL upper reference limit.

*Significant (α = 0.005 after correction for multiple comparisons).

### Initial treatment

Only one patient with pure sensory GBS received no treatment while the remaining 99% (120/121) of patients received at least one treatment. IVIg was used as the first treatment in 88% (107/121) of the cohort, while 10% (12/121) were initially treated with PE and 1% (1/121) received corticosteroids. In patients with severe GBS, PE was numerically more frequently used as the initial treatment (13%) compared to patients with mild GBS (7%) but there was no statistical difference between the two groups regarding the choice of first treatment (*p* = 0.36). The median delay from symptom onset to initial treatment was 5.5 days (IQR 3–11). There was no statistically significant difference in time from symptom onset to treatment between patients with severe and mild GBS (5 vs. 6 days, *p* = 0.20).

### Sequential treatments

**Figure 1:**
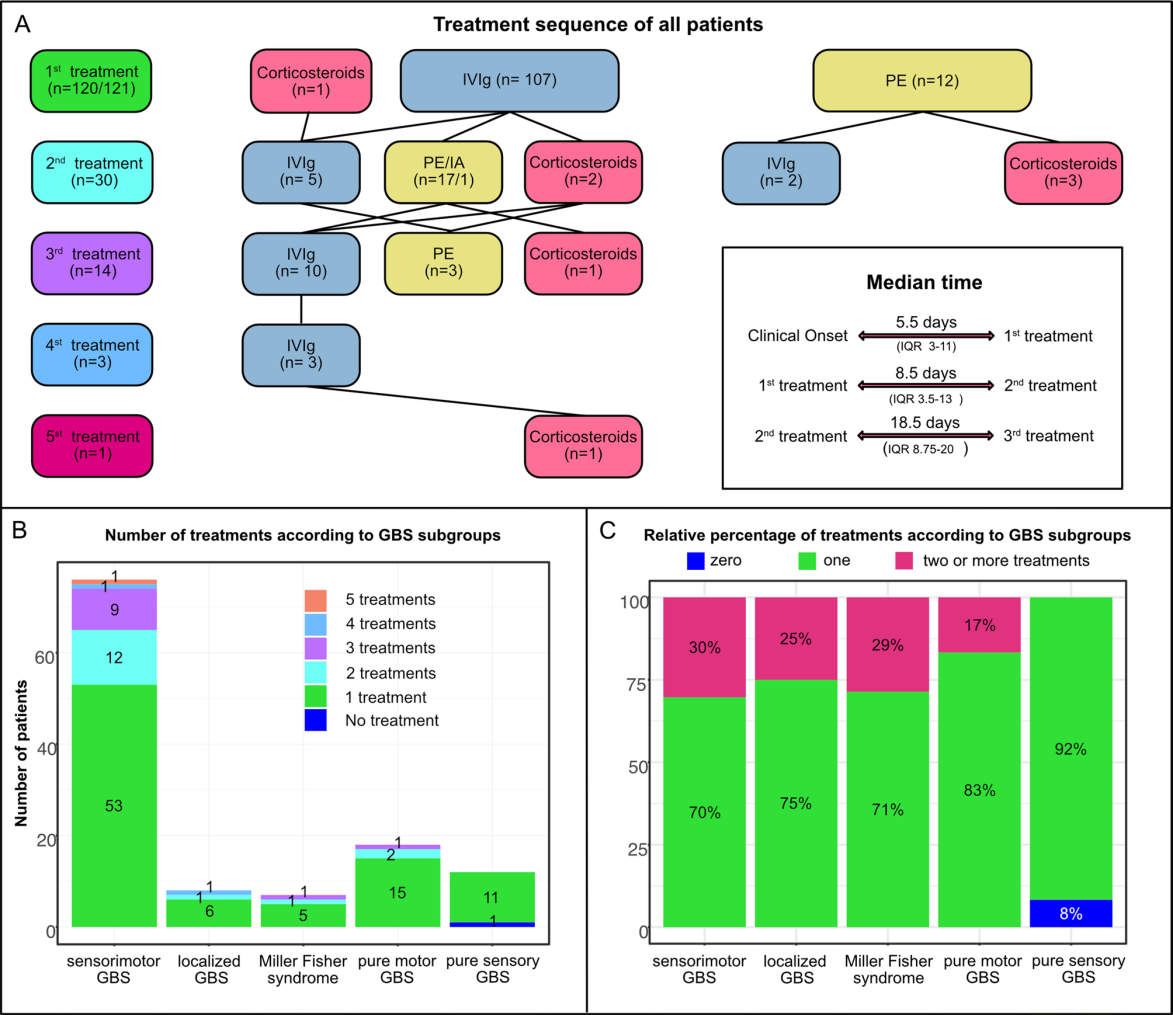
Number of treatments. (**A**) Treatment sequence of all patients included in the study. Rows correspond to sequential treatments (first to fifth) and the overall number of patients per sequential treatment is depicted within the squared boxes on the left column. Type of treatment is indicated by text and color and the respective number of patients receiving a specific treatment modality is shown within the squared boxes. The median time from symptom onset to treatment as well as between treatments with IQR in brackets is shown in the field on the lower right. (**B**) Absolute number of received treatments according to GBS subgroups. (**C**) Relative percentages of received treatments (grouped as zero or one vs. two or more) according to GBS subgroups.

25% (30/121) of the cohort received a second treatment after a median of 15.5 days (IQR 6–18) and 12% (14/121) received 3 or more treatments. The choice of the second treatment was PE in 57% (17/30) of patient, immunoadsorption (IA) in 3% (1/30), IVIg in 23% (7/30) and corticosteroids in 17% (5/30). The detailed treatment sequence as well as the absolute number of treatments and relative percentage for each GBS subgroups are shown in Fig. 1. The reason for a second treatment was persistent clinical deterioration in 47% (14/30), lack of clinical improvement after the initial therapy in 30% (9/30) and a TRF in 13% (4/30). The remaining 10% (3/30) of patients received a second treatment despite clinical improvement.

**Figure 2:**
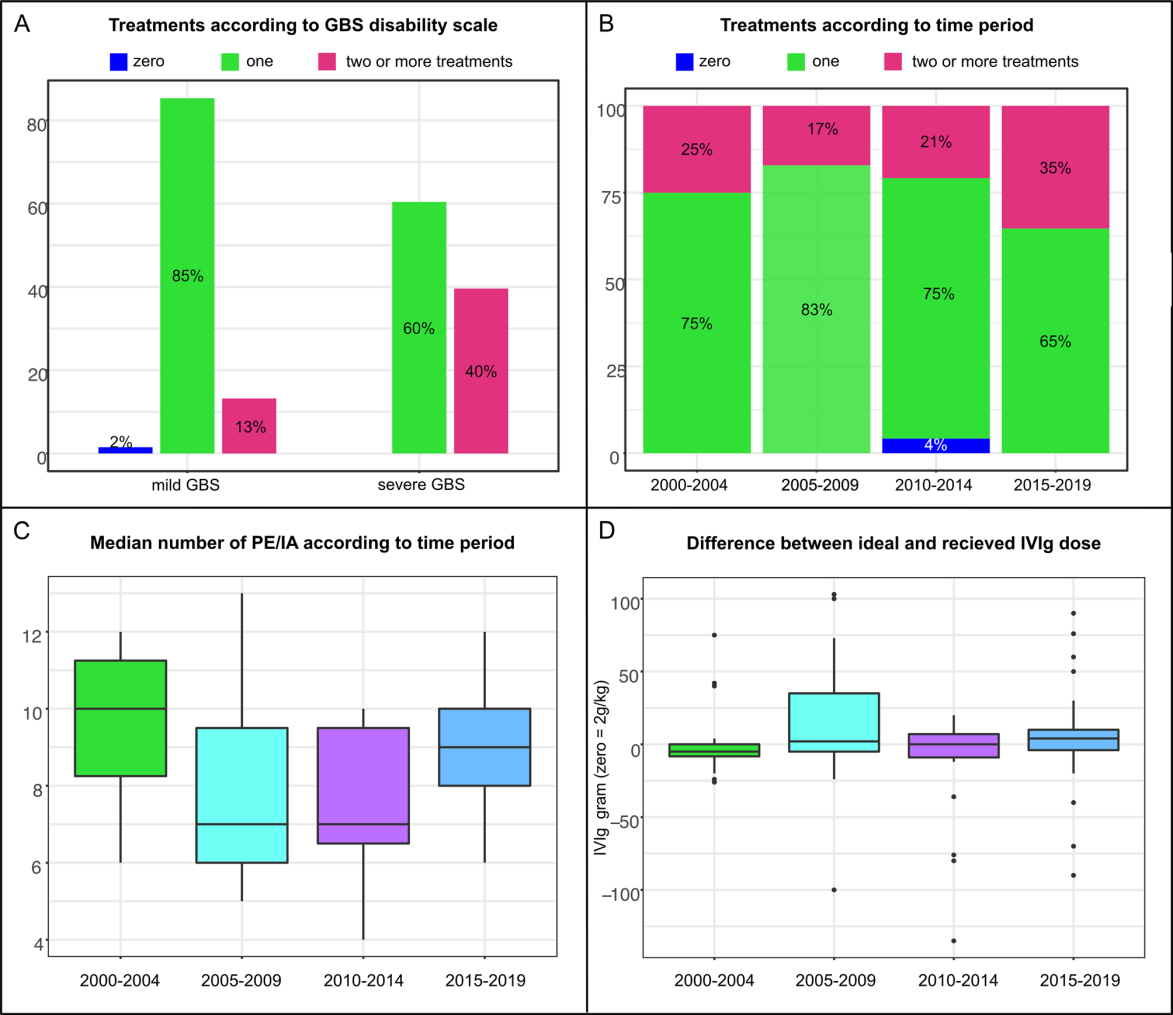
Treatment details. (**A**) Number of treatments according to clinical severity of GBS with mild GBS defined as a GBS disability scale of 0–2 and severe GBS defined as a GBS disability scale of 3 or higher (i.e., non-ambulatory). (**B**) Number of treatments for each time period of diagnosis grouped into 5-years intervals between 2000 and 2019. (**C**) Boxplots of the median number of plasma exchanges (PE) or immunoadsorption (IA; one patient) for each time period of diagnosis grouped into 5-years intervals between 2000 and 2019. The lower and upper hinges correspond to the first and third quartiles and the lower and upper whisker extend from the hinge to the smallest and largest value no further than 1.5 times the interquartile range from the hinge. Outliers are plotted individually as black points. (**D**) Boxplots of the median difference between the ideal IVIg dose (defined as 2 g per kilogram bodyweight) and the actual received dose. Boxplots are shown for all IVIg treatments in each time period of diagnosis grouped into 5-years intervals between 2000 and 2019. Definition of hinges and whisker is the same as in C and outliers are plotted individually as black points.

The multivariate logistic regression model showed that presence of severe GBS at nadir had a significant adjusted effect on receiving more than one treatment. Specifically, the estimated odds ratio for patients with severe GBS compared to patients with mild GBS to receive more than one treatment was 4.22 (95%CI 1.36–13.10, *p* = 0.01). No significant adjusted effects were found for age at onset, sex, GBS subgroups and date of onset (5-year intervals between 2000 and 2019). Figure 2 provides corresponding bar plots for the number of treatments according to clinical severity (Fig. 2A) and the time period of onset grouped into four 5-year intervals between 2000 and 2019 (Fig. 2B).

### Duration, dosage and number of treatment courses

Of 127 IVIg treatments, 7 were stopped prematurely because of clinical deterioration and switched to treatment with PE, while 2 had to be discontinued because of adverse events. Consequently, 100 patients completed 118 IVIg treatments. Information about dose and body weight was available in 117. In the majority of courses (81%), IVIg was given over 5 days, while only 5% received IVIg over 3 days, 9% over 4 days, and 5% over more than 5 days (up to 9 days). The median difference between the ideal IVIg dose (2 g/kg body weight) and the actually received dose was 0 g (IQR − 6 g to 9 g) but with a wide range (− 135 g to 103 g). Figure 1 shows box plots for the difference between the ideal and the actually received IVIg dose for each 5-year interval time period between 2000 and 2019.

The median number of plasma exchanges or immunoadsorptions per patient was 8 (IQR 6–10) with a range between 4 and 13. PE/IA was performed over a median of 13 days (IQR 9.5–19, range 4–49). The general linear model showed that neither GBS severity, age, sex, date of onset or GBS subgroup had a significant adjusted effect on the number of plasma exchanges. Figure 1 shows boxplots for the number of PE/IA for each 5-year interval time period between 2000 and 2019.

### Outcome

The median time from symptom onset to the last follow-up was 56 days (IQR 36.5–92, range 18–356). Of all patients, 88% (106/121) had a good outcome (i.e., a GBS disability scale < 3).

The remaining 12% (15/126) had a poor outcome but no patient of the study cohort died. Older age (OR 1.05, 95%CI 1.00–1.11, *p* = 0.05) and electrophysiological axonal subtype according to Rajabally’s criteria^15^ (OR 13.48, 95%CI 1.13–160.93, *p* = 0.04) were associated with poor outcome while sex and clinical GBS subtype were not. 53% (8/15) of patients with poor outcome received more than one treatment compared to 21% (22/106) of patients with good outcome (*p* = 0.01). One patient with severe ventilator-depended GBS, who was excluded from the analysis due to insufficient data, died after transfer to another hospital; thus, mortality of all 129 screened patients was 0.8%.

**Figure 3:**
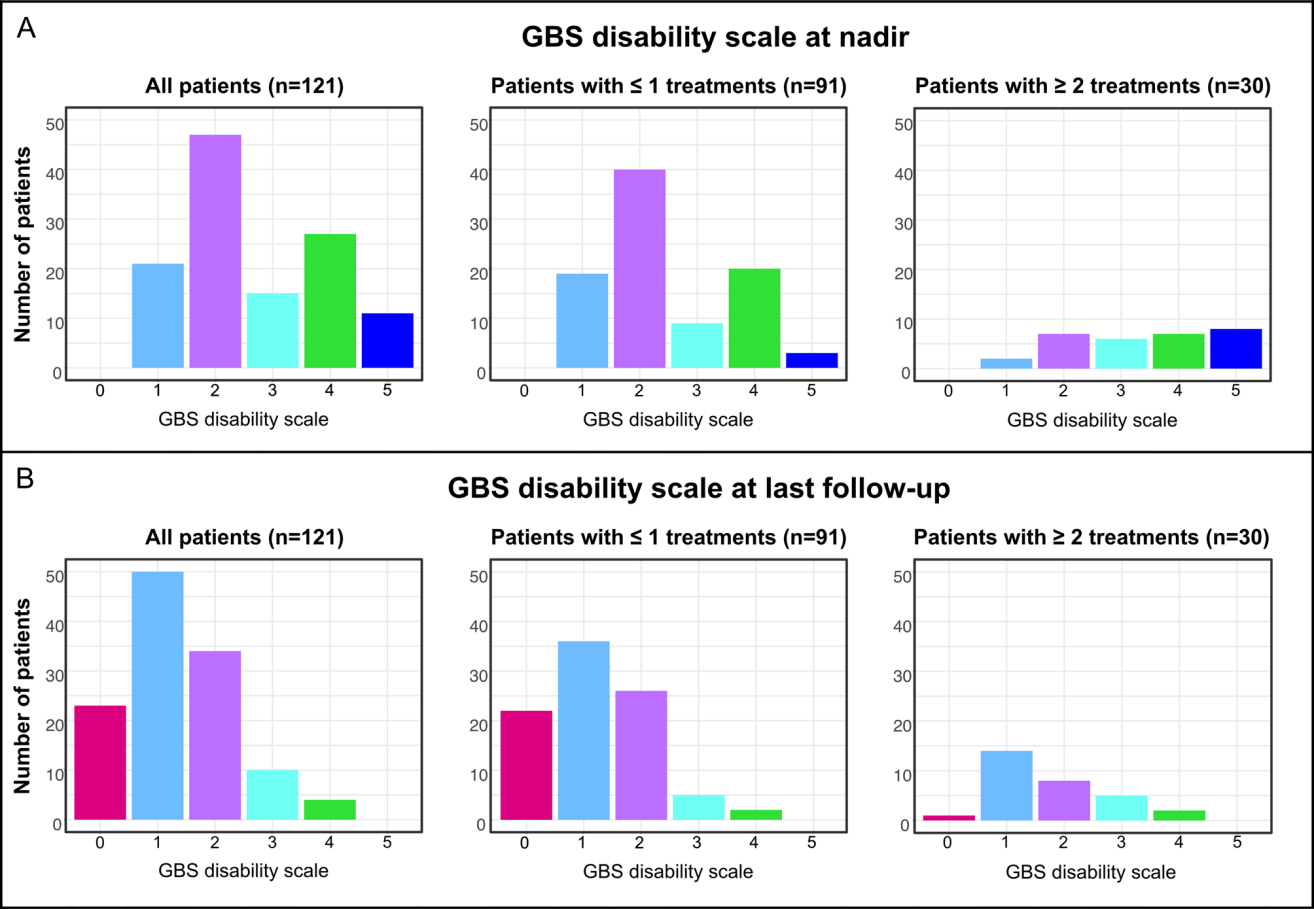
GBS disability scale. Number of patients in each GBS disability scale category (ranging from 0 to 6 with higher scores indicating more severe disease) at nadir (**A**) and last follow-up (**B**). Barplots are shown for all patients and for patient groups according to number of treatments (zero or one vs. two or more treatments). The GBS disability score of 6 is not displayed as no patient died during the study.

An improvement of the GBS disability scale from nadir to last follow-up was seen in 79% (96/121) of patients. 16% (4/25) of patients who failed to improve received more than one treatment compared to 27% (26/96) of patients who improved by at least one point (p = 0.25). In patients with severe GBS, the multivariate logistic regression showed no significant adjusted effect of receiving multiple treatments treatment on clinical improvement between nadir and last follow-up (OR 1.30, 95%CI 0.31–5.40, *p* = 0.72). Nor was there an effect of the parameters GBS subgroup, age, sex or time to first treatment. Figure 3 shows the number of patients in each GBS disability scale category at nadir and last follow-up.

## Discussion

In this study, we retrospectively analyzed treatment practice in a large cohort of patients with GBS over 20 years at a tertiary care center in Austria. The main finding was that a substantial number of patients with severe GBS received multiple treatments without a significant effect on clinical improvement between nadir and last follow-up visits. Moreover, almost all patients with GBS received at least one immunomodulatory treatment regardless of clinical severity or subtype and this did not change over the last two decades.

Compared to the previous literature, the number of patients receiving any treatment in our study was very high (99%). The largest study to date, which evaluated treatment practice of GBS from 2012 and 2017 in 1023 patients included in the International GBS Outcome Study (IGOS), reported that 92% of patients received any immunomodulatory treatment^9^. The higher proportion of treated patients in our study could probably reflect a tertiary care center bias with less stringent economic hospital policies. However, the number of treated patients remained high over the course of 20 years in our cohort regardless of clinical severity of subtype suggesting that clinical decision making also did not change over time despite the limited data regarding patients with mild or localized GBS and MFS. While one trial reported a better outcome for two vs. zero plasma exchanges in patients with mild GBS^18^, the majority of studies that investigated the effect of PE or IVIg^3,4,6^ included only patients with severe (i.e., non-ambulatory) GBS. Regarding MFS, most patients have a good recovery even without treatment^19^ and IVIg and PE have not been investigated in randomized trials in this subgroup^20^.

The most frequently used first-line therapy in our cohort was IVIg (88%), which is also in line with the number reported in the IGOS cohort (84%), suggesting that IVIg availability was stable over the last two decades and is the preferred treatment for most patients due to its easier accessibility. Corticosteroids were used in a small number of patients as an adjunctive or sequential treatment despite largely missing evidence for a positive effect^21^, although a mild effect of i.v. methylprednisolone in combination with IVIg was proposed to be associated with additional short-term improvement^22^. However, the assumingly main reasons for the use of corticosteroids in clinical practice are considerations of differential diagnoses, especially in patients with localized variants or when there is a possibility of acute-onset CIDP.

In our cohort, 25% of patients received more than one treatment without a significant change of this number over the study time period. Sequential treatments were mostly given because of clinical improvement or continuous decline and only seldomly because of TRFs. Multiple treatments were mainly (but not exclusively) applied in the subgroup of patients with severe GBS, in which 40% received more than one treatment. However, treating patients with more than one therapy had no significant effect on the rate of clinical improvement between nadir and last follow-up in patients with severe GBS. Our findings are of course only based on observational, retrospective data and therefore limited by the heterogeneity of data and the unstandardized time to the last follow-up. Nevertheless, our data argue against a strong effect of multiple treatments on short-term outcome. To investigate whether repeated treatments had negative effects due to adverse events—as suggested by the recent randomized study for IVIg^8^—was beyond the scope of our study. However, given the comparable rate of side effects of IVIg and PE it is currently hard to justify any sequential treatment in patients with GBS, regardless of clinical severity or assumed prognosis. Patients with TRFs may be one possible exception to this rule, since an immunological flare-up after initial treatment response is discussed for this patient subgroup^23^.

PE was more often used compared to IVIg as second line treatment. Indeed, in a subset of these patients, the initial IVIg treatment was prematurely stopped and the therapy switched to PE, a practice not only possibly mitigating the effect of IVIg by washing them out^24^ but not supported by current data, which rather show a comparable effect of the two treatment modalities^6^. However, the generally higher obstacles to immediately start PE compared to IVIg might falsely insinuate a therapeutic advantage of PE. Some patients also received up to five treatments, a practice which likely is of limited effect given the rather long time to treatment initiation, where an effect of immunomodulatory treatments in GBS is questionable^2^.

We also found that the number of PEs performed at our center was markedly higher with a median of 8 than in clinical trials or in IGOS, where commonly 5 PEs were performed^4^. Moreover, the median PE number differed slightly between time periods (Fig. 1C) and the number of days over which PE was performed was also heterogenous. Evidence regarding the optimal number of PE is limited, with only one study reporting that 4 PEs were better than 2 in patients with moderate GBS and that 6 PEs were not superior to 4 PEs in patients with severe, ventilator-dependent GBS^18^. Since PE was performed more often as a second line treatment in patients without clinical improvement or continuous deterioration in our cohort, a possible explanation is that clinical improvement was not seen immediately in these more severely affected patients, negatively impacting the decision to stop treatment.

Recommended IVIg dosing is generally 2 g/kg bodyweight given over 5 days. While in general these dose recommendations were followed in our cohort, in a small subset of patients the received dose differed significantly from this recommendation (Fig. 2D), and almost a fifth of patients did not receive IVIg over the recommended 5 days. Small deviations from the ideal dose might have been due to availability of IVIg formulations or calculation errors; however, in the small number of patients with markedly different doses than recommended, the decision was presumably made based on the clinical disease course, clearly deviating from the available evidence and current recommendations^1,2^.

The strength of our study is the long time period of 20 years over which patients were retrospectively analyzed with treatment and clinical data extracted from patient charts. This enabled us to analyze treatment practice in a large cohort of unselected patients reflecting clinical practice outside of specialized neuromuscular wards. Furthermore, patients were treated by a large number of different physicians on different wards and intensive care units and the data therefore reflect also the heterogeneity in training and experience of physicians in everyday clinical scenarios regarding the management of GBS.

There are some limitations of this study. First, our study included only adult patients and the results are not applicable to pediatric GBS. Second, the time to last follow-up was heterogenous with a wide range, thus limiting the overall outcome assessment and precluded any analysis regarding a possible effect of repeated treatments on time to recovery. Additionally, due to the retrospective design, we were not able to assess a potential effect of multiple treatments on ongoing progression or length of hospital stay, parameters that could be more sensitive than the sole comparison of GBS disability between nadir and last follow-up. Moreover, while we assessed the number of PE and the start of treatment, we could not extract detailed information (e.g., volume per exchange). Future studies should investigate whether PE methodology changed over time. Regarding IVIg dosing the ideal dose was retrospectively calculated from patient reported body weight and not from actually measured body weight. However, outside of clinical studies, this also reflects real-world clinical practice.

In conclusion, we showed that over the last two decades, nearly all patients with GBS received any kind of treatment regardless of clinical severity or subtype. A considerable proportion of patients received multiple treatments without a significant effect on clinical improvement between nadir and last follow-up. Moreover, we found that treatment practices were heterogenous regarding number of PE, IVIg dosing and duration. Future studies should investigate whether the subgroup of patients with TRFs benefits from repeated treatments and explore new therapeutic add-on options.

## Data Availability

Data can be made available from the corresponding author upon reasonable request and after approval from the ethics review board at the Medical University of Vienna.

## Abbreviations

CI: Confidence interval
CIDP: Chronic inflammatory demyelinating polyneuropathy
GBS: Guillain Barré syndrome
IA: Immunoadsorption
IVIg: Intravenous immunoglobulins
IQR: Interquartile range
MFS: Miller Fisher syndrome
MRC: Medical Research Council
OR: Odds ratio
PE: Plasma exchange
TRF: Treatment related fluctuations
